# Editorial: Advances in the development and application of deep eutectic solvents

**DOI:** 10.3389/fchem.2023.1258718

**Published:** 2023-07-27

**Authors:** Maria Luisa Di Gioia, Ana Rita C. Duarte, Manoj B. Gawande

**Affiliations:** ^1^ Department of Pharmacy, Health and Nutritional Sciences, University of Calabria, Arcavacata di Rende, Italy; ^2^ Nova School of Science and Technology, Faculty of Science and Technology, New University of Lisboa, Caparica, Portugal; ^3^ Department of Industrial and Engineering Chemistry, Institute of Chemical Technology, Jalna, Maharashtra, India

**Keywords:** green chemistry, reactive deep eutectic solvents, deep eutectic solvent (DES), green solvents, therapeutic deep eutectic solvents

## 1 Introduction

The discovery of deep eutectic solvents (DES) was a major breakthrough in green and sustainable chemistry. Notably, DES distinctive properties have increased their applications in different research areas, attracting attention as a means of achieving sustainable chemistry. DES are excellent solvents and/or catalysts used in organic synthesis, in extracting bioactive compounds from natural matrices, and in solubilizing gases.

The Research Topic “*Advances in the development and application of Deep Eutectic Solvents*,” comprehends a Research Topic of nine articles (seven original research papers, and two reviews), highlighting the most recent and relevant applications of DESs.

Nowadays, environmental issues and the energy crisis are some of the most important challenges faced by researchers. DESs physically dissolve gases, and are employed for gas capture and gas separation in various industries (Paludetto Pelaquim et al., 2021). Three of the published papers in the Research Topic dealing with this striking aspect. The proper choice of the hydrogen-bond donor as the constituent of the DES, is a fundamental issue in solubility of gas as it affects the absorption capacity. Alhadid et al. evaluate the feasibility of exploiting nonionic phenolic alcohols based DESs as solvents for carbon dioxide (CO_2_) capture applications. The nonionic DESs show higher stability and ability in terms of CO_2_ solubility respect to ionic Liquids (ILs) and ionic DESs already proposed in the literature.

In order to assess the capability of DESs for such tasks, the thermodynamic modelling of CO_2_ solubility in DESs has been pursued by several researchers. Parvaneh et al. investigate the performance of the Perturbed Chain- Statistical Associating Fluid Theory (PC-SAFT). The authors developed the largest data bank, up to date, of CO_2_ solubility in DESs, consisting of 109 different DESs over wide ranges of temperatures and pressures. The work shows the PC-SAFT model to be very valuable for screening and feasibility studies to select potential DESs among the innumerable options today available.

The challenge of reduction greenhouse gas emissions can also be addressed through the conversion of CO_2_ into high value-added chemicals or fuels. This recycling technology is presented by Zhang et al. which design four new kinds of natural deep eutectic solvents (NADES) to be employed as the co-electrolyte in converting CO_2_ into methanol via electro-enzymatic processes. The serine and glycerol DES shows high CO_2_ solubility, high electro-catalytic activity as well as high methanol production, resulting in a promising approach in the CO_2_ capture research field.

Recently, a plethora of extraction techniques using NADES has arisen as eco-friendly alternatives to conventional extraction procedures, especially for the recovery of bioactive compounds from waste. The Research Topic includes three original research papers and one review dealing with this application. Vieira et al. provide an important contribution, suggesting the possibility of performing a fractionated extraction of bioactive compounds from rosemary waste, using a biphasic system composed by two immiscible DESs. Each phase of this system selectively extract the compounds of interest, selecting them by polarity. Another paper from Batista et al. focuses on the sustainable extraction of proteins from renewable sources. In particular, they propose a straightforward extraction approach using a citric acid:xylitol:water NADES for the recovery of biocompatible collagen from skin waste, resulting from the blue shark fishing industry. The procedure avoids any pre-treatment of the raw materials, and significantly improves the extraction yields, when compared to the traditional procedures.

The use of DESs as medium for protein extraction and purification is also addressed in the review by Bowen et al. DESs maintain the biological and/or functional activity of the extracted proteins, and improve their stability. Nevertheless, selection of hydrogen-bonding donor (HDB), the presence of water during DES formation, and the structure–function relationship existing between the extracted proteins and DESs should be taken into account.

In some cases, protein solubilization processes can be hindered by the DES viscosity which is mainly related to hydrogen bonds. The addition of cosolvents to choline chloride (ChCl)-based DESs is more and more investigated for reducing their high viscosities. Engelbrecht et al. contribute to the Research Topic with a manuscript reporting a computational investigation aiming to explain experimental observations made on excess molar enthalpies in pseudo-binary mixtures of a ChCl/ethylene glycol DES with water or methanol. The molecular dynamics simulation reveals an intriguing difference in the interaction modes of the two cosolvents with the DES chloride anion. As a result, they draw the conclusion that various intermolecular interactions in the resultant DES/cosolvent may favor one application over another. DESs are investigated also in organic synthesis where they find application as solvents, and often also as reagents and/or catalysts. In this context, the manuscript by Lončarić et al. reports on the synthesis of a particular class of pharmacologically active heterocyclic compounds, such as thiazolidine-2,4-dione derivatives, using DES that act as both solvents and catalysts. The ChCl: *N*-methylurea DES affords the pure products in good to high yields via a simple precipitation by adding water. Most importantly, the recyclability of the system confirms the greenness of the procedure.

The second review published in this article Research Topic by Buhrman et al. aims to provide evidence and stimulate researchers to a more in-depth knowledge of the occurrence of NADES as a third liquid phase in living organisms as proposed for the first time by Choi et al., (2011). Their hypothesis was that NADES may play an important role in solubilizing, storing, and transporting poorly water-soluble metabolites in living cells, adjusting the water content of plants, and protecting cells when in harsh conditions (Choi et al., 2011). Buhrman et al. provide a critical and organized overview of all the data that supports the assumption that there is a link between accumulation of anthocyanin flavonoids in highly concentrated inclusions and the presence of NADES as an inert solvent. These types of mixtures could represent an important aspect of the natural environment in cells.
